# Explainable deep-learning framework: decoding brain states and prediction of individual performance in false-belief task at early childhood stage

**DOI:** 10.3389/fninf.2024.1392661

**Published:** 2024-06-28

**Authors:** Km Bhavna, Azman Akhter, Romi Banerjee, Dipanjan Roy

**Affiliations:** ^1^Department of Computer Science and Engineering, IIT Jodhpur, Karwar, Rajasthan, India; ^2^Cognitive Brain Dynamics Lab, National Brain Research Centre, Manesar, Gurugram, India; ^3^School of AIDE, Center for Brain Science and Applications, Indian Institute of Technology (IIT), Jodhpur, India

**Keywords:** decoding of brain states, graph neural networks, theory of mind, false-belief task, pain networks

## Abstract

Decoding of cognitive states aims to identify individuals' brain states and brain fingerprints to predict behavior. Deep learning provides an important platform for analyzing brain signals at different developmental stages to understand brain dynamics. Due to their internal architecture and feature extraction techniques, existing machine-learning and deep-learning approaches are suffering from low classification performance and explainability issues that must be improved. In the current study, we hypothesized that even at the early childhood stage (as early as 3-years), connectivity between brain regions could decode brain states and predict behavioral performance in false-belief tasks. To this end, we proposed an explainable deep learning framework to decode brain states (Theory of Mind and Pain states) and predict individual performance on ToM-related false-belief tasks in a developmental dataset. We proposed an explainable spatiotemporal connectivity-based Graph Convolutional Neural Network (Ex-stGCNN) model for decoding brain states. Here, we consider a developmental dataset, *N* = 155 (122 children; 3–12 yrs and 33 adults; 18–39 yrs), in which participants watched a short, soundless animated movie, shown to activate Theory-of-Mind (ToM) and pain networs. After scanning, the participants underwent a ToM-related false-belief task, leading to categorization into the pass, fail, and inconsistent groups based on performance. We trained our proposed model using Functional Connectivity (FC) and Inter-Subject Functional Correlations (ISFC) matrices separately. We observed that the stimulus-driven feature set (ISFC) could capture ToM and Pain brain states more accurately with an average accuracy of 94%, whereas it achieved 85% accuracy using FC matrices. We also validated our results using five-fold cross-validation and achieved an average accuracy of 92%. Besides this study, we applied the SHapley Additive exPlanations (SHAP) approach to identify brain fingerprints that contributed the most to predictions. We hypothesized that ToM network brain connectivity could predict individual performance on false-belief tasks. We proposed an Explainable Convolutional Variational Auto-Encoder (Ex-Convolutional VAE) model to predict individual performance on false-belief tasks and trained the model using FC and ISFC matrices separately. ISFC matrices again outperformed the FC matrices in prediction of individual performance. We achieved 93.5% accuracy with an F1-score of 0.94 using ISFC matrices and achieved 90% accuracy with an F1-score of 0.91 using FC matrices.

## 1 Introduction

Decoding of cognitive states from the brain activity, or simply the “brain decoding” has emerged as one of the most active research areas because of its potentially wide-ranging implications in medical and therapeutic engineering fields (Santhanam et al., 2006; Hou et al., 2022). Due to its noninvasive approach and considerable spatial and temporal resolution, functional magnetic resonance imaging (fMRI) is commonly used to decode cognitive states. Traditional fMRI techniques use generalized linear models to predict regional brain activity based on specific behavioral tasks or cognitive states that a participant performs or experiences. This approach can mistakenly be interpreted in reverse–assuming specific activation patterns indicate definite cognitive states (Poldrack, 2011; Zhang et al., 2021). However, this is often inaccurate, as patterns of activity by different tasks and states can be overlapping. It's been suggested that reverse inference can be more reliably applied through brain decoding methods, where spatiotemporal activity is used to predict cognitive states under various conditions (Poldrack, 2006; Zhang et al., 2021). Across the wide literature, the terms “cognitive states,” “brain states,” and “task-states” have been used more or less synonymously. To avoid any confusion, we mostly stick with the term “cognitive states,” with few instances of “brain states.” By both, we mean the state of the brain during specific cognitive processes or behavioral tasks.

Although there have been significant improvements in brain decoding about specific cognitive states, there exist also genuine knowledge gap.Previous studies (Haxby et al., 2001; Li and Fan, 2019; Wang et al., 2020) attempted to generate models that could decode brain states across a wide range of behavioral domains. Meta-analytic methodologies have been utilized for multi-domain decoding (Bartley et al., 2018). However, meta-analyses face several limitations, such as inconsistent samples across cognitive domains, publication bias favoring positive results, and inflated effect sizes from small studies (Dubben and Beck-Bornholdt, 2005; Alamolhoda et al., 2017; Lin, 2018; Zhang et al., 2021). In areas with limited research, these issues can bias decoding analyses and lead to incorrect inferences (Lieberman and Eisenberger, 2015; Lieberman et al., 2016; Wager et al., 2016). An alternate method is to overcome these biases by training linear classifiers on activation maps acquired from a group of individuals (Poldrack et al., 2009; Bzdok et al., 2016; Varoquaux et al., 2018; Zhang et al., 2021).

A few studies have employed Deep Neural Network (DNN) models, such as Convolutional Neural Networks (CNNs), which are efficient, scalable, and can differentiate patterns without requiring manual features. 3d CNN-based models are shown to be efficient at decoding states of the brain across multiple domains of stimulus processing (Wang et al., 2020). However, there are some limitations of DNNs; training DNN architectures with fully connected layers is challenging, particularly in neuroimaging applications, because of many free parameters and a limited number of labeled training data. As a result, these architectures tend to overfit the data and exhibit poor out-of-sample prediction (Zhang et al., 2021). Secondly, though DNN performs admirably with grid-like inputs in Euclidean space, such as (natural) images, the distance in Euclidean space may not adequately represent the functional distance between different parts of the brain (see similar; Rosenbaum et al., 2017). Instead, geometric deep-learning (DL) methods, such as graph convolutional networks (GCNs), would better suit non-Euclidean data types, such as brain networks (Zhang and Bellec, 2019; Zhang et al., 2021).

Critically, extant DL approaches do not exploit the dynamic spatiotemporal characteristics of brain activity during naturalistic movie-watching paradigms. These paradigms provide a promising pathway to examine brain dynamics across a diverse spectrum of realistic human experience(s) and provide a rich context-dependent array of cognitive states and sub-states to be investigated with the help of machine learning (ML) models (Simony and Chang, 2020). Also, DNN models that decode brain data from the developmental period are lacking. We believe that modeling stimulus-evoked activity patterns of children and adolescents from naturalistic movie-watching paradigms can more effectively characterize states across multiple cognitive domains, especially, those of higher-order cognition like Theory of Mind (ToM).

To address these challenges, we developed a novel spatiotemporal graph convolutional neural network model (stGCNN) that inputs functional connectivity (FC) and inter-subject functional connectivity (ISFC), derived from BOLD time-series data from key brain regions. This model effectively captures the spatiotemporal dynamics of brain activity to differentiate between brain activation patterns associated with two cognitive states: the perception of others' pain and Theory of Mind (ToM) processing in children and adolescents. Our study aimed to a) develop an explainable spatiotemporal decoding model to classify brain activation patterns using connectivity features, FC and ISFC, during movie watching in children, adolescents, and adults (control), and b) use contributing features from the previous model to predict individual performance on false-belief tasks.

**Figure 1:**
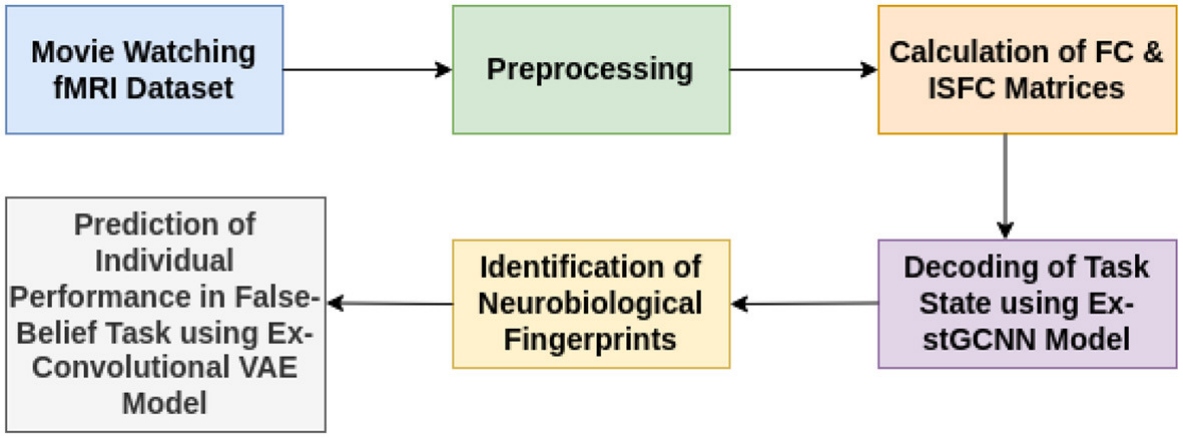
Illustrative overview of end-to-end explainable deep-learning framework for decoding of cognitive states and prediction on performance of false-belief task-based pass, fail, and inconsistent groups.

The stGCNN model is based on a graph Laplacian-based model that models the brain as a graph treating region-of-interest (ROI) as nodes and their connectivity as edges. The proposed explainable spatiotemporal connectivity-based graph convolutional neural network (Ex-stGCNN) model accurately decodes time courses during which participants experienced a particular cognitive state while watching the movie (Refer to Figure 1). The proposed model could extract features from non-Euclidean data and process graph-structured signals. We used FC, which reflects inter-regional correlations arising from a mixture of stimulus-induced neural processes, intrinsic neural processes, and non-neuronal noise, and ISFC, which isolates stimulus-dependent inter-regional correlations by modeling the BOLD signal of one brain on the other brain's exposed to the same stimulus (Simony et al., 2016), as feature set to train the proposed model. As a result, we achieved an average of 94 % accuracy with an F1-Score of 0.95. We applied the SHAP (SHapley Additive exPlanations) method for explainability and finally identified neurobiological brain features that contributed the most to the prediction. Then we implemented the unsupervised Explainable Convolutional Variational Autoencoder model (Ex-Convolutional VAE) to predict individual performance in false-belief tasks in which FC and ISFC matrices were used as feature sets. We obtained 90 % accuracy using FC matrices as a feature set with an F1-Score of 0.92% and 93.5% accuracy with an F1-score of 0.94 using ISFC matrices. To validate the results, we implemented Five-fold cross-validation. We have made a comparison with previously employed models and found that our Convolutional Variational Auto Encoder (CVAE) model gave the best prediction accuracy. The final challenge we address here is one of the most interesting questions in neuroscience related to identifying neurobiologically meaningful features at the individual participant level that predict their performance in the cognitive task. To our knowledge, no previous DL classification study in mentalization tasks has investigated neurobiologically interpretable spatiotemporal brain features that robustly predict Theory of Mind task performance in children, adolescents along with adults, without feature engineering. This framework not only decoded brain states for groups of different developmental ages and adults and highly imbalanced datasets with high accuracy from short-time course data but also predicted individual performance in false-belief tasks to classify participants into pass, fail, and inconsistent groups independent of their behavioral performance ratings. Based on our theoretical model, we predict that social cognition networks [comprised of bilateral Temporoparietal Junction (LTPJ and RTPJ), Posterior Cingulate Cortex (PCC), Ventral and Dorsal-medial Prefrontal Cortex (vmPFC and dmPFC), and Precuneus] feature prominently in the prediction of cognitive performance in children and adolescents during early development.

## 2 Materials and methods

### 2.1 Participants and fMRI preprocessing

**Figure 2:**
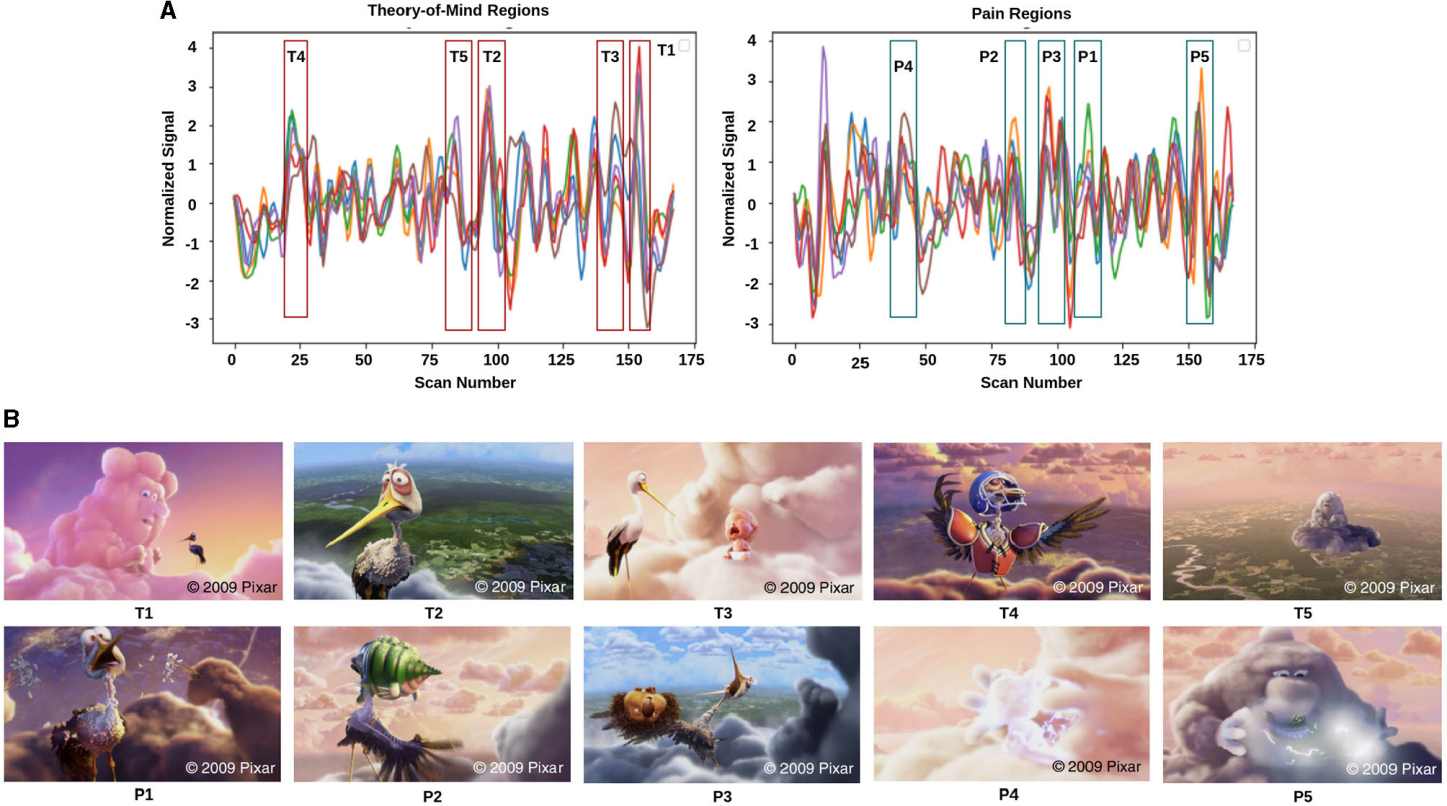
Movie demonstration: **(A)** response magnitude that evoked maximum activation in ToM and pain networks. **(B)** depicts the movie scenes with higher activation. *T*_*i*_∈[*T*_1_, *T*_2_, *T*_3_, *T*_4_, *T*_5_] is representing ToM scenes, and *P*_*i*_∈[*P*_1_, *P*_2_, *P*_3_, *P*_4_, *P*_5_] is representing pain scenes with higher activation.

To develop models for investigating Theory-of-Mind and Pain networks across developmental stages, we analyzed a dataset of 155 early childhood to adult participants, available on the OpenfMRI database. (The childhood group consisted of 122 participants aged 3–12-yrs (mean age = 6.7 yrs, SD = 2.3, 64 females), complemented by 33 adults (mean age = 24.8 yrs, SD = 5.3, 20 females) (Astington and Edward, 2010; Richardson et al., 2018; Bhavna et al., 2023). Participants who participated in the study were from the surrounding neighborhood and brought in a signed permission form from a parent or guardian. The approval for data collection was given by the Committee on the Use of Humans as Experimental Subjects (COUHES) at the Massachusetts Institute of Technology. In this experiment, participants watched a soundless short animated movie of 5.6 min named “Partly Cloudy” (Refer to Figure 2). Using a dataset that included developmental age groups (3–12 yrs) and individuals in adulthood opened the opportunity to propose a framework for the decoding of cognitive states that could analyze complex brain dynamics in the early childhood stage and contextualize these findings from the perspective of adult brains. After scanning, six explicit ToM-related questions were administered for the false-belief task to identify the correlation between brain development and behavioral scores in ToM reasoning. Each child's performance on the ToM-related false-belief task was assessed based on the proportion of questions answered correctly out of 24 matched items (14 prediction items and 10 explanation items). Based on the outcome of these explicit false-belief task scores, the participants were categorized into three classes: Pass (5–6 correct answers), inconsistent (3–4 correct answers), and fail (0–2 correct answers) (Reher and Sohn, 2009; Astington and Edward, 2010; Jacoby et al., 2016; Richardson et al., 2018). A 3-Tesla Siemens Tim Trio scanner at the Athinoula A. Martinos Imaging Center at MIT was used to collect whole-brain structural and functional MRI data (For head coil details, see Richardson et al., 2018). Children under 5 used one of the two custom 32-channel head coils: younger (*n* = 3, M(s.d.) = 3.91(0.42) yrs) or older (*n* = 28, M(s.d.) = 4.07(0.42) yrs) children; all other participants used the standard Siemens 32-channel head coil. With a factor of three for GRAPPA parallel imaging, 176 interleaved sagittal slices of 1 mm isotropic voxels were used to get T1-weighted structural images (FOV: 192 mm for child coils, 256 mm for adult coils). The whole brain was covered by 32 interleaved near-axial slices that were aligned with the anterior/posterior commissure and used a gradient-echo EPI sequence sensitive to BOLD contrast to capture functional data (EPI factor: 64; TR: 2 s, TE: 30 ms, flip angle: 90) (Richardson et al., 2018). All functional data were upsampled in normalized space to 2 mm isotropic voxels. Based on the participant's head motion, one TR back, prospective acquisition correction was used to modify the gradient locations. The dataset was preprocessed using SPM 8 and other toolboxes available for Matlab (Penny et al., 2011), which registered all functional images to the first run image and then registered that image to each participant's structural images (Astington and Edward, 2010). All structural images were normalized to Montreal Neurological Institute (MNI) template (Burgund et al., 2002; Cantlon et al., 2006). The smoothing for all images was performed using a Gaussian filter and identified Artifactual timepoints using ART toolbox (Astington and Edward, 2010; Whitfield-Gabrieli et al., 2011).

### 2.2 fMRI data analysis and extraction of feature sets

**Figure 3:**
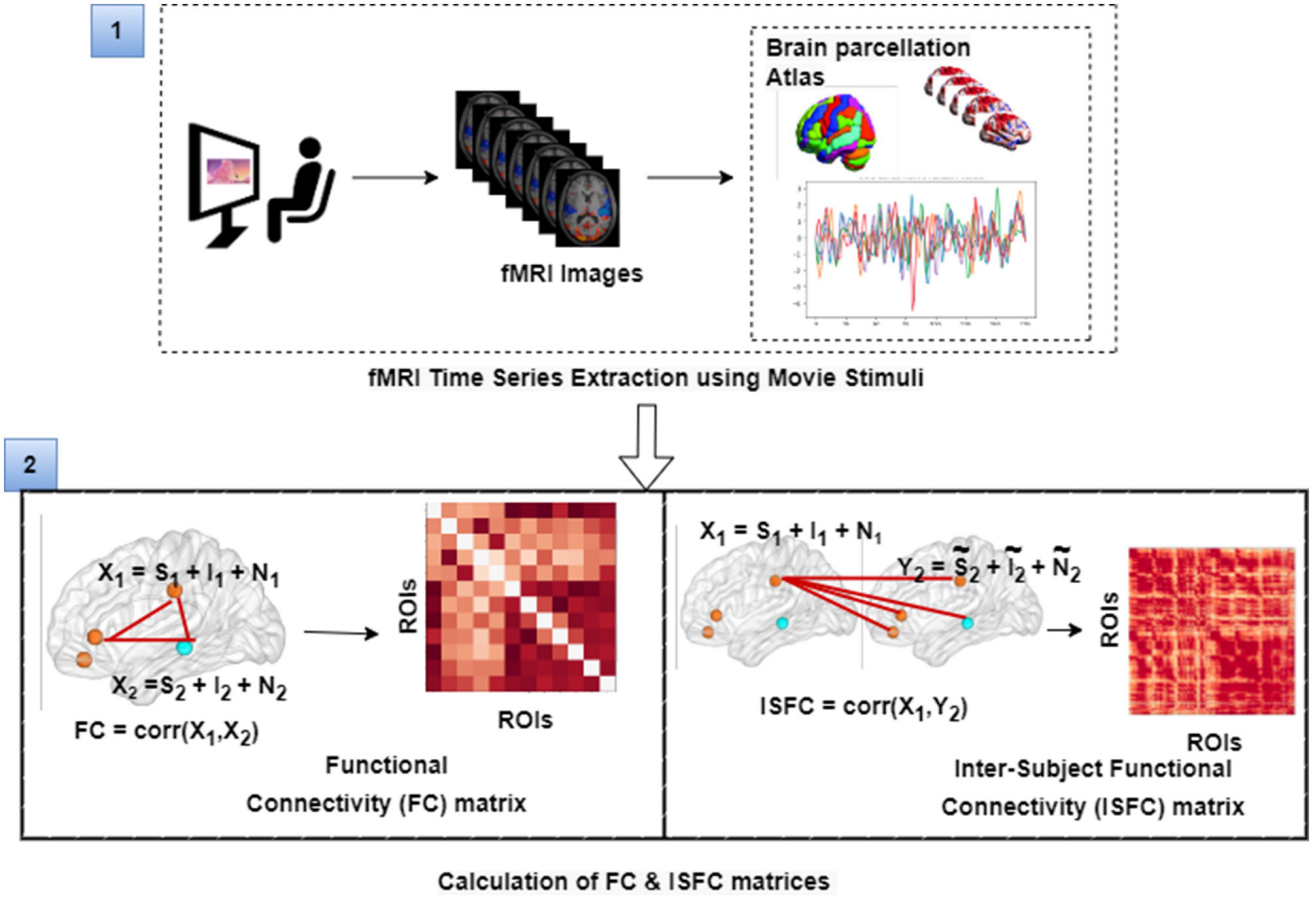
The figure illustrates the calculation of FC and ISFC matrices from movie-watching fMRI data that has been further used as input for decoding of cognitive state and prediction of individual performance in false-belief task purpose. Here, *S* represents task-evoked brain activity; *I* represents intrinsic brain activity; *N* represents noise.

The film features two main characters, Gus, a cloud, and his stork friend Peck, experiencing bodily sensations (notably physical pain) and complex mental states (such as beliefs, desires, and emotions). The depiction of these experiences–categorized into pain scenes and Theory of Mind (ToM) scenes–serves to investigate the viewers' brain networks that are activated during the understanding of physical and emotional states. These scenes effectively highlight the developmental changes in neural circuits involved in percieving others' physical sensations and mental conditions. Based on previous studies, we selected twelve regions of interest (ROIs) six from the Theory of Mind (ToM) network including bilateral Temporoparietal Junction (LTPJ and RTPJ), Posterior Cingulate Cortex (PCC), Ventral and Dorsal-medial Prefrontal Cortex (vmPFC and dmPFC), and Precuneus, and six from the pain network comprising bilateral Middle Frontal Gyrus (LMFG and RMFG), bilateral Insula, and bilateral Secondary Sensory Cortex (LSSC and RSSC) (Mazziotta et al., 1995, 2001; Baetens et al., 2014). A spherical binary mask of 10 mm radius was applied around the peak activity within these ROIs during specific scenes, from which we extracted time-series signals as detailed in Table 1. The selected scenes were chosen for their ability to elicit the strongest responses in the ToM and pain networks at specific time points, as illustrated in Figure 2 and Table 2. Similar to the the main study of the dataset, we extracted short-duration time-courses corresponding to peak events–five each from ToM and pain scenes, yielding a total of ten time-courses and 168 time-points. Finally, we calculated FC and ISFC as separate feature sets (Refer to Figure 3).

1. **Resting state-functional connectivity:** To calculate functional connectivity matrices for each participant for different time courses, we calculated Pearson's correlation between the average time series BOLD signals that were extracted from each of the spherical brain regions.
2. **Computation of inter-subject functional correlations:** ISFC has been used to characterize brain responses related to dynamic naturalistic cognition in a model-free way (Simony et al., 2016; Kim et al., 2018; Lynch et al., 2018; Demirtaş et al., 2019). ISFC assesses the region-to-region neuronal coupling between subjects instead of intra-subject functional connectivity (FC), which measures the coupling inside a single participant (Hasson et al., 2004; Nastase et al., 2019). ISFC delineates functional connectivity patterns driven by extrinsic time-locked dynamic stimuli (Hasson et al., 2004; Simony et al., 2016; Xie and Redcay, 2022). We calculated ISFC to check the coupling between ROIs across all the subjects.

**Table 1:** ToM and pain brain regions and corresponding MNI-coordinated for extracting time-series signal.

**ToM regions**			**Pain regions**		
**Sr. No**.	**ROIs**	**MNI-Coordinates (X,Y,Z)**	**Sr. No**.	**ROIs**	**MNI-Coordinates (X,Y,Z)**
1	Posterior cingulate cortex (PCC)	0, -52, 18	1	Right Middle Frontal Gyrus (RMFC)	36, 38, 40
2	Left temporoparietal junction (LTPJ)	-46, -68, 32	2	Left Middle Frontal Gyrus (LMFC)	–36, 38, 40
3	Right temporoparietal junction (RTPJ)	46, -68, 32	3	Left Interior Insula (LII)	–40, 22, 0
4	Ventromedial Prefrontal cortex (vmPFC)	4, 48, -4	4	Right Interior Insula (RII)	39, 23, –4
5	Precuneus	0, –49, 40	5	Left secondary sensory cortex (LSSC)	–39, –15, 18
6	Dorsomedial prefrontal cortex (dmPFC)	–10, 58, 24	6	Right secondary sensory cortex (RSSC)	39, –15, 18

**Table 2:** Description of movie-clip events with higher activation for ToM and pain networks.

**ToM clips description**		**Pain clips description**	
**Sr. No**.	**Description**	**Sr. No**.	**Description**
T1	Peck flies away to happy cloud	P1	Gus pulls porcupine spines from Peck's head
T2	Peck caught gazing at happy clouds	P2	Alligator biting Peck
T3	Baby crying, then happy	P3	Peck tossing porcupine
T4	Peck dons gear to show why he left	P4	Cloud makes animals (lightning)
T5	Pan from happy clouds to lonely cloud (Gus)	P5	Gus makes alligator (lightning)

### 2.3 Decoding of states using explainable spatiotemporal connectivity based graph convolutional neural network

We hypothesized that stimulus-driven brain features, ISFC, could decode cognitive states (ToM and Pain) more accurately than FC features. To check our hypothesis, we implemented the Explainable Spatiotemporal connectivity-based Graph Convolutional Neural Network (Ex-stGCNN) approach to classify states evoked during watching stimuli. In previous work (Richardson et al., 2018), the author applied reverse correlation analysis to average response time series to determine points of maximum activation in ToM and pain networks. We accordingly selected five-time courses (>8 sec), from each ROI, of maximum activation in ToM and Pain networks (total of ten-time courses) (Refer to Table 2). Then, we extracted time-series and converted it into a 2D matrix T*N format for each individual where T = no. of time steps, and *N*= no. of regions. We calculated FC matrices of size 12*12 for each time course (10 matrices for each individual) and the same for ISFC matrices. Finally, we trained our Ex-stGCNN model in two different ways: (a) using FC matrices and (b) using ISFC matrices.

#### 2.3.1 Proposed architecture

**Figure 4:**
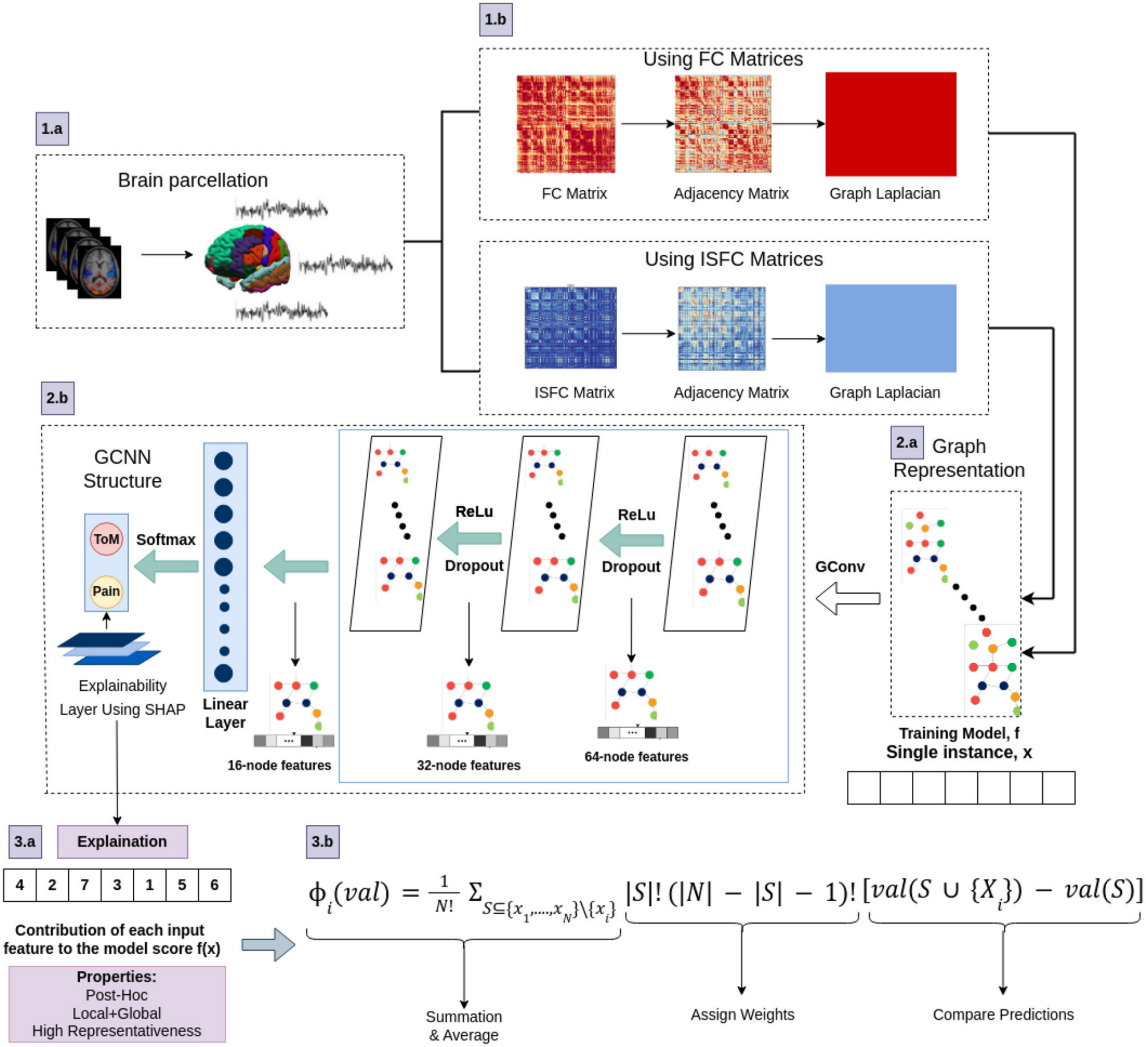
The architecture of proposed Ex-stGCNN model. Firstly, FC and ISFC matrices are converted into adjacency matrices and then into graph Laplacian. Finally, the graph representation of these matrices is provided as input to the model for training. We trained this proposed model using FC and ISFC matrices separately. The SHAP approach is used to extract neurological brain fingerprints to check the contribution of each brain region in the prediction.

Using PyTorch and PyTorch Geometric, the proposed model was developed in which, for every node, the Scalable Hypothesis tests (tsfresh) algorithm was used for statistical feature extraction (Kipf and Welling, 2016; Fey and Lenssen, 2019; Paszke et al., 2019; Saeidi et al., 2022). Using the FRESH algorithm concept (Christ et al., 2016), the tsfresh algorithm combined the elements from the hypothesis tests with the feature statistical significance testing. By quantifying p-values, each created feature vector was separately analyzed to determine its relevance for the specified goal. Finally, the Benjamini-Yekutieli process determined which characteristics to preserve (Benjamini and Yekutieli, 2001). We utilized node embedding methods to extract the high-level features associated with each node. We implemented Walklets and Node2Vec node embedding algorithms to observe node attributes from graph (Grover and Leskovec, 2016; Perozzi et al., 2017). Three convolutional layers were used in the proposed Ex-stGCNN model, where every layer had 300 neurons. The Rectified Linear Unit (ReLU) and batch normalization layers were implemented between each CNN layer to speed convergence and boost stability. After each CNN layer, dropout layers were applied to decrease the inherent unneeded complexity and redundant computation of the proposed multilayer Ex-stGCNN model. The final graph representation vector was calculated by applying a global mean pooling layer (Refer to Table 3 and Figure 4).

**Table 3:** Table shows an implementation of the proposed GCNN model architecture, where O = no. of task, N is = input size, *F*_*i*_∈[*F*_1_, *F*_2_, *F*_3_] = no. of filters at *i*_*th*_ graph convolutional layer, K = polynomial order of filters.

**Proposed GCNN model**							
**Layer (Type)**	**Maps (Filters)**	**Edges**	**Polynomial order**	**Pooling size**	**Activation**	**Weights**	**Bias**
Input	1	$\Sigma_{i=1}^{\left(N-1\right)}i$	-	-	-	-	-
Convolution (C1)	*F*_1_ (16)	$\Sigma_{i=1}^{\left(N-1\right)}i$	K	-	ReLU	1*F_1_*K	N*F_1_
Max-pooling (M1)	*F*_1_ (16)	$\Sigma_{i=1}^{\left(N-1\right)}i$	-	2	-	-	-
Convolution (C2)	*F*_2_ (16,32)	$\Sigma_{i=1}^{\left(N/2-1\right)}i$	K	-	ReLU	*F*_1_*F_2_*K	*N*/2*F_2_
Max-pooling (M2)	*F*_2_ (16,32)	$\Sigma_{i=1}^{\left(N/2-1\right)}i$	-	2	-	-	-
Convolution (C3)	*F*_3_ (16,32,64)	$\Sigma_{i=1}^{\left(N/4-1\right)}i$	K	-	ReLU	*F*_2_*F_3_*K	*N*/4*F_3_
Max-pooling (M3)	*F*_3_ (16,32,64)	$\Sigma_{i=1}^{\left(N/4-1\right)}i$	-	2	-	-	-
Flatten	-	-	-	-	-	-	-
Fully connected (Softmax)	-	-	-	-	Softmax	*N*/4*N/4*F_3_*O	O

The mathematical formation of the proposed architecture is as follows: A graph G = (V, E) consists of a set of nodes (*v*_1_, *v*_2_, ...., *v*_*n*_) and edges such that *E*_*ij*_ = (*v*_*i*_, *v*_*j*_)∈*E* and *E*⊆*V*×*V*. Here, the edge has two end-points, i.e., *v*_*i*_ and *v*_*j*_, which are connected through e and also refer as adjacent nodes. For developing Graph Neural Network *f*(*X, A*), where *X* is representing feature matrix of the nodes in the graph and *A* is indicating adjacency matrix, we considered spatiotemporal connectivity-based multilayer Graph convolutional neural network using Equation (1) that indicated forward propagation rule (Kipf and Welling, 2016):


(1) $$\begin{array}{l} H^{l+1}=\sigma \left(\;D^{-1/2}\;D^{-1/2}H^{l}W^{l}\right) \end{array}$$

Where *A* denotes adjacency matrix i.e. *A*+*I* for undirected graph G, whereas *D*_*ii*_ = Σ_*j*_ *A*_*ij*_ and *W*^*l*^ are a layer-specific trainable weight matrix. σ(.) denotes an activation function, such as the *ReLU*(.) = *max*(0, ). *H*^*l*^∈*R*^*N*×*D*^ is the matrix of activation at lth layer.

#### 2.3.2 Spectral based GCN

We consider spectral convolutions on graphs (GCNs), which are defined as a signal's multiplication *x*∈*R*^*N*^ by a filter *g*_θ_ = *diag*(θ) using Equation (2). The graph Laplacian's eigen-decomposition in the Fourier domain was calculated via spectral GCNs using the Laplacian matrix (Kipf and Welling, 2016).


(2) $$\begin{array}{l} g_{\theta }⋆x=U_{g_{\theta }}U^{T}x \end{array}$$

Where U denotes eigenvector matrix of normalized graph Laplacian *L* = *I*−*D*^−1/2^*AD*^−1/2^ = *UΛU*^*T*^, and *U*^*T*^*x* denotes transformation from graph Fourier to a signal x. *g*_θ_ represents function of the eigenvalues of L.

Due to the multiplication with eigenvector matrix U is *O*(*N*^2^), which is a complete matrix with n Fourier functions, this procedure is computationally expensive. To avoid quadratic complexity, the authors in Yan et al. (2019) suggested the ChebNet model, which ignores the eigendecomposition by utilizing Laplacian's learning function. The filter *g*_θ_ is estimated via the ChebNet model using Chebyshev polynomials of the diagonal matrix of eigenvalues, as illustrated in Equation (3):


(3) $$\begin{array}{l} g_{\theta^{{\;}^{\prime }}}⋆x\approx \overset{K}{\underset{k=0}{\sum }}\theta_{k}^{{\;}^{\prime }}T_{k}\tilde{\Lambda } \end{array}$$

Where diagonal matrix Λ∈[−1, 1] and $\tilde{\Lambda }=\frac{2}{\lambda_{max}}\Lambda -I$. λ_*max*_ indicates largest eigenvalue of L, θ′∈*R*^*K*^ = vector of Chebyshev coefficients. Chebyshev polynomial is denoted as *T*_*k*_(*x*) = 2*xT*_*k*−1_(*x*)−*T*_*k*−2_(*x*) with *T*_0_(*x*) = 1 and *T*_1_(*x*) = *x*. We calculated convolution of signal x with $g_{\theta^{\prime }}$ filter using Equation (4) (Kipf and Welling, 2016):


(4) $$\begin{array}{l} g_{\theta^{{\;}^{\prime }}}⋆x\approx \overset{K}{\underset{k=0}{\sum }}\theta_{k}^{{\;}^{\prime }}T_{k}\left(\tilde{L}\right)x \end{array}$$

Where $\tilde{L}=\frac{2}{\lambda_{max}}L-I$, and λ_*max*_ defines greatest eigenvalue of L.

#### 2.3.3 Training and testing

We trained our model on FC matrices and ISFC matrices separately. In current study, the dataset was divided using an 80:20 ratio, and this process was carried out in a random yet controlled manner to ensure non-overlapping subsets (Rácz et al., 2021). The following steps were undertaken to split the data:

1. Random Shuffling: The dataset *D* consisting of *N* subjects was randomly shuffled to eliminate any inherent ordering.
2. Splitting: The shuffled dataset was then divided into training and testing sets using an 80:20 ratio. Specifically, the first 80% of the data (after shuffling) formed the training set *D*_*train*_, and the remaining 20% formed the testing set *D*_*test*_.

Mathematically, this can be represented as follows:

- Let *D* = *d*_1_, *d*_2_, ..., *d*_*N*_ be the dataset with *N* subjects.
- After shuffling, the dataset becomes $D^{\prime }=d_{1}^{{\;}^{\prime }},d_{2}^{{\;}^{\prime }},...,d_{N}^{{\;}^{\prime }}$, where *D*′ is a permutation of *D*.
- The training set *D*_*train*_ is defined as $D_{train}=\left\{d_{1}^{\prime },d_{2}^{\prime },\ldots ,d_{\left\lfloor 0.8N\right\rfloor }^{\prime }\right\}$.
- The testing set *D*_*test*_ is defined as $D_{test}=\left\{d_{\left\lfloor 0.8N\right\rfloor +1}^{{\;}^{\prime }},d_{\left\lfloor 0.8N\right\rfloor +2}^{{\;}^{\prime }},...,d_{N}^{{\;}^{\prime }}\right\}$.

To ensure robustness and avoid any potential bias from a single random split, we repeated this process 10 times, each time with a new random shuffle of the dataset. This procedure ensures that the subsets are non-overlapping across different splits, and the performance metrics reported in our results are averaged over these 10 independent splits.

We used learning rate = 0.001, dropout = 0.65, and weight decay = 0.0, patience = 3 (Saeidi et al., 2022). As batch size is one of the most crucial hyperparameters to tune, a set of batch size values was also considered. This study was implemented using an Adam (Adaptive Moment Estimation) optimizer with batch sizes of B = [16, 32, 64] across 100 epochs. For the final prediction, we used the Softmax activation function using Equation (5):


(5) $$Softmax({\hat{y}}_{i})=\frac{exp({\hat{y}}_{i})}{\Sigma_{i=1}^{O}exp({\hat{y}}_{i})}$$

Where, ŷ_*i*_∈[ŷ_1_....ŷ_*O*_] represents predicted probability of *i*_*th*_ task. Additionally, the optimization function was run using cross-entropy loss using Equation (6):


(6) $$Loss=-\Sigma_{i=1}^{O}y_{i}\log({\hat{y}}_{i})+\frac{\rho }{2N_{P}}∥W{∥}^{2}$$

Where *y*_*i*_ indicates targetted tasks, W represents network parameters, *N*_*P*_ represents no. of parameters, and ρ indicates weight decay rate. To validate the results, we also implemented five-fold cross-validation.

#### 2.3.4 Identification of neurobiological features and analysis using five-fold cross validation and leave-one-out methods

Deep learning models, particularly those involving deep neural networks, suffer from a significant black-box problem because they operate in ways that are not easily interpretable. This complexity arises due to the multiple layers and numerous parameters involved in these models. Gradient-based approaches, decomposition methods, and surrogate methods are some techniques developed to explain existing GNNs from various perspectives (Yuan et al., 2022). In the existing studies, the perturbation-based approaches were implied to identify the link between input characteristics and various outputs (Ying et al., 2019; Schlichtkrull et al., 2020; Yuan et al., 2021). However, in decoding applications, none of these techniques can guarantee the discovery of plausible and comprehensible input characteristics from a neuroscience standpoint.

In this study, the SHAP (SHapley Additive exPlanations) feature diagnostic technique was used to determine the neurological features that contributed most to Decoding of cognitive states, also referred to as dominant brain regions. The SHAP value for a feature is calculated as the average marginal contribution of that feature across all possible feature subsets. Specifically, the SHapley value for feature *i* is computed by summing the contributions of *i* in each subset *S*, where *S* does not contain *i*. The contribution of *i* is measured by the difference in model predictions when *i* is included and when it is excluded from *S*, appropriately weighted to account for the different sizes of feature subsets. We applied SHAP approach using Equation (7).


(7) $$\phi_{i}(v)=\frac{1}{N!}\sum_{S\subseteq x_{i},..,x_{N}\setminus x_{i}}|S|!(|N|-|S|-1)![val(S\cup (X_{i}))-val(S))]$$

Where: N represents all possible subsets, *S* is a subset of features that does not include feature *i*, *S* represents the number of features in subset *S*, *N* is the total number of features, *val*(*S*∪(*i*)) is the model prediction when feature *i* is added to subset *S*, and *val*(*S*) is the model prediction for subset *S* without feature *i*. To reduce the chance of bias and report low variance, we implemented a five-fold cross-validation method to evaluate the models performance (precision, recall, accuracy, F1-score) and the leave-one-out method to see performance of the model at individual level.

### 2.4 Prediction of individual performance in false-belief task using explainable convolutional variational autoencoder model

In a previous study (Richardson et al., 2018), the authors conducted a ToM-based false-belief task for the 3-12 yrs age group after fMRI scanning and divided all participants into three groups, i.e., pass, fail, and inconsistent, based on their performance. The previous studies (Li et al., 2019a,b; Finn and Bandettini, 2021) reported an association between brain signals and behavioral scores in resting state and during movie-watching stimuli. We hypothesized that FC and ISFC between brain regions could predict individual performance in false-belief tasks. To check our hypothesis, we used a developmental dataset with 122 participants in which the age range varies from 3–12 yrs, comprising 84 passers, 15 failures, and 23 inconsistent performers. We conducted this analysis in three ways: (a) including all 12 brain regions; (b) including dominant brain regions (Total of 8); and c) including only six ToM regions. After decoding cognitive states from FCs and ISFCs, we identified brain regions that contributed the most to prediction also referred as dominant brain regions. There were three dominant regions from ToM networks and three dominant regions from pain networks, overall six regions from ISFC-based analysis and six regions from FC-based analysis. As there was an overlap between the set of regions across analysis-type, we ended up with 8 dominant regions in total. Finally, we proposed an Explainable Convolutional Variational Auto-Encoder model (Ex-Convolutional VAE), in which we provided FC and ISFC matrices of each participant as input and performed prediction of individual performance in false-belief tasks and categorized them into pass, fail, or inconsistent groups. Ex-Convolutional VAE model included two components: (1) an encoder, which transforms the original data space (X) into a compressed low-dimensional latent space (Z), and a decoder, which reconstructs the original data by sampling from the low-dimensional latent space. (2) Use of latent space for prediction using ADAM optimizer.

**Figure 5:**
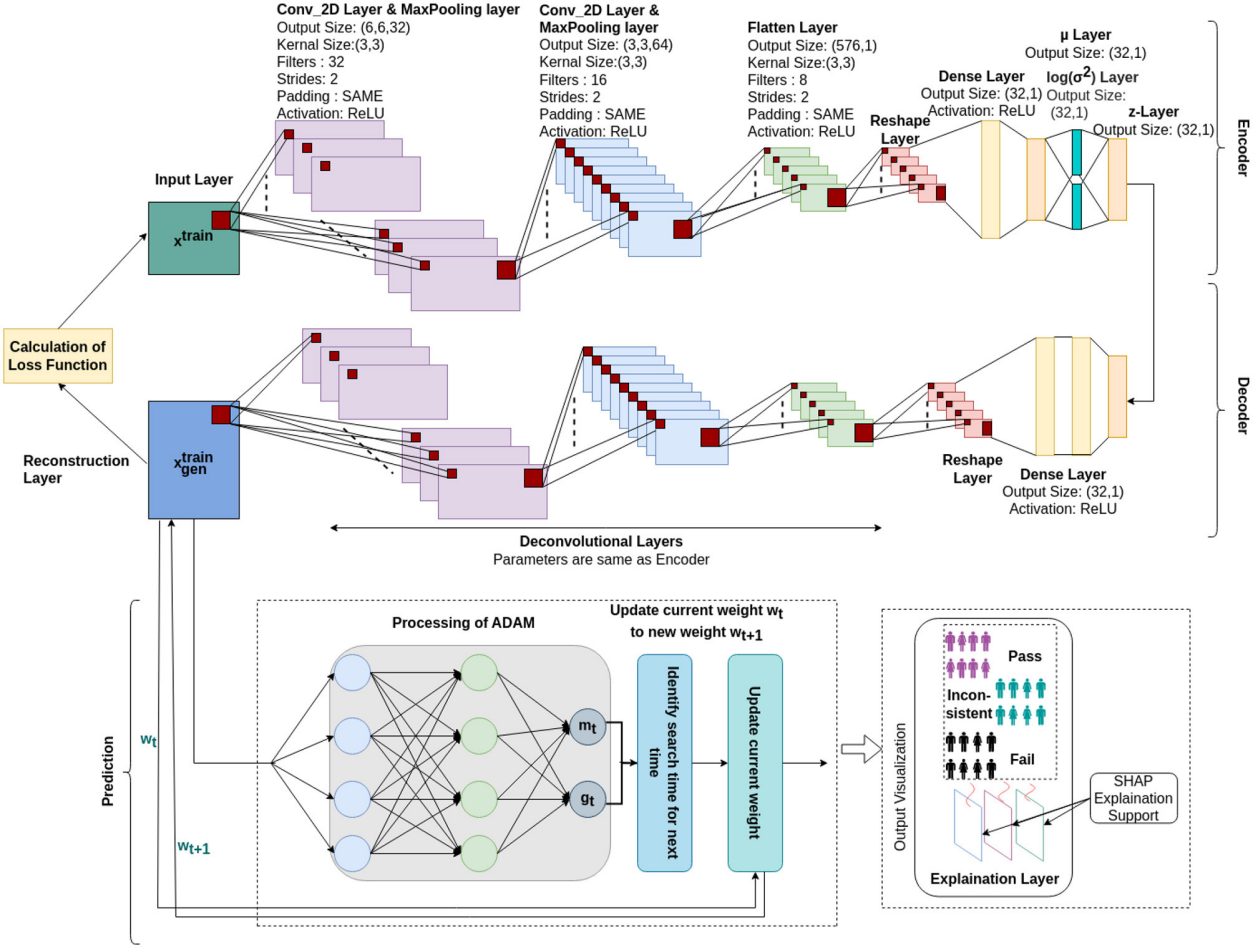
Architecture of proposed Ex-Convolutional VAE model for predicting false-belief task-based pass, fail, and inconsistent groups. The proposed architecture first used the 2D convolutional approach to create a 32-dimensional latent space and trained the prediction model using the ADAM optimization approach (a 32-D latent space was used to train the model). The SHAP approach is used for the explainability of the proposed model.

The proposed Ex-Convolutional VAE model included 2D convolutional layers with ReLU activation function followed by flattening and dense layers with ReLu activation (kernal:3, filters: 32, strides: 2, epoch: 50, latent dimension: 32, no. of channel: 1, batch size: 128 for training Ex-Convolutional VAE and 32 for prediction, padding: SAME, activation function: ReLU for training and sigmoid for prediction) (Refer to Figure 5 and Table 4). The dense layer was used to produce an output of the mean and variance of the latent distribution. Using the reparameterization technique, the sampling function used mean and log variance to sample from latent distribution. The decoder architecture included a dense layer followed by a resampling layer, and 2D transposed convolutional layers with ReLU activation function. We used mean squared error (MSE) and Kullback-Leibler (KL) techniques to calculate the loss. The reason for using KL was its ability to regularize learned latent distribution to be close to standard normal distribution. We used trained Ex-Convolutional VAE Latent space for training prediction model with ADAM optimization technique. We performed the prediction using the sigmoid activation function and binary-cross entropy to calculate the loss function (epochs: 50).

**Table 4:** Table shows proposed architecture of ex-convolutional VAE model and the parameters with values that have been used on it.

**Encoder**				**Decoder**		
**Layer (Type)**	**Output shape**	**Param #**	**Connected to**	**Layer (Type)**	**Output shape**	**Param #**
encoder_ input (InputLayer)	(12, 12, 1)	0		z_sampling (InputLayer)	(32, 1)	0
conv2d_28 (Conv2D)	(6, 6, 32)	320	encoder_input[0][0]	dense_55 (Dense)	(576, 1)	19008
conv2d_29 (Conv2D)	(3, 3, 64)	18, 496	conv2d_28[0][0]	reshape_14 (Reshape)	(3, 3, 64)	0
flatten_15 (Flatten)	(576, 1)	0	conv2d_29[0][0]	conv2d_transpose_28 (Conv2DTranspose)	(6, 6, 64)	36, 928
dense_54 (Dense)	(32, 1)	18, 464	flatten_15[0][0]	conv2d_transpose_29 (Conv2DTranspose)	(12, 12, 32)	18, 464
z_mean (Dense)	(32, 1)	1, 056	dense_54[0][0]	decoder_output (Conv2DTranspose)	(12, 12, 1)	289
z_log_var (Dense)	(32, 1)	1,056	dense_54[0][0]			
Total params: 39, 392, Trainable params: 39392				Total params: 74, 689, Trainable params: 74, 689		

#### 2.4.1 Proposed approach

In a variational autoencoder model, the encoder produces latent space from a given input while the decoder produces output from this latent space. The decoder inferences that the latent vectors have a normal probability distribution; the parameters of that which are the mean and variance of the vectors, calculated using Equation (8) (Lee et al., 2022):


(8) $$\begin{array}{l} p\left(x|z\right)=N\left(x|f_{\mu }\left(z\right),f_{\sigma }{\left(z\right)}^{2}*I\right) \end{array}$$

Where, x represents original data space, z represents compressed low-dimensional latent space, *p*(*x*|*z*) indicates assumed probability distribution, *f*_μ_(*z*) indicates the mean of latent space, and $f_{\sigma }{\left(z\right)}^{2}*I$ represents variance of latent space. In this particular circumstance, the marginal likelihood estimation technique can be used to the best of its ability to maximize the log-marginal likelihood of the model using Equation (9):


(9) $$\begin{array}{l} log\;p\left(x\right)=log\Sigma_{z}p\left(x|f_{\mu }z,f_{\sigma }{\left(z\right)}^{2}*I\right)p\left(z\right) \end{array}$$

However, it is challenging to maximize the log-marginal likelihood in this form. As a result, we develop variational inference, which simplifies the range of possible outcomes by approximating the posterior probability distribution (Zhang et al., 2018). An approximately normal probability distribution is an appropriate approximation for the posterior probability distribution. Applying the learning method may be challenging if the input has a high dimension (Lee et al., 2022). To resolve this, the inferred probability distribution is calculated as a function of x using Equations (10, 11).


(10) $$\begin{array}{l} q\left(z\right)=N\left(\mu_{q},\sigma_{q}^{2}\right) \end{array}$$

(11) $$\begin{array}{l} q\left(z|x\right)=N\left(\mu_{q}\left(x\right),\Sigma_{q}\left(x\right)\right) \end{array}$$

Where *q*(*z*) is inferred normal probability distribution, and *q*(*z*|*x*) is its expression as function of x. Finally, we can obtain the latent vector z by combining the mean value with the product of the inferred normal distribution and the variation. The term “reparameterization trick” refers to the process used to add a new parameter or feature expressed by Equation (12):


(12) $$\begin{array}{l} z=\mu \left(x\right)+\sigma \left(x\right)*\epsilon ,\epsilon \sim N\left(0,1\right) \end{array}$$

Where z represents latent space, and ϵ represents a normally distributed random variable.

The Kullback–Leibler divergence is used to calculate loss function by updating weights and biases that calculate the difference between the actual posterior distribution and inferred distribution (Kullback and Leibler, 1951; Kingma and Welling, 2013) using the Equation (13).


(13) $$\begin{array}{l} \;D_{KL}(q(z)∥p(z|x))\\ =D_{KL}(q(z|x)∥p(z))+log\;p(x)-E_{z\sim q(z)}[log\;p(x|z)]) \end{array}$$

Using the above equation, the log-marginal likelihood of the decoder can be expressed by Equation (14).


(14) $$\begin{array}{l} log\;p(x)=E_{z\sim q(z|x)}[log\;p(x|z)]-D_{KL}(q(z|x)∥p(z))\\ \;+D_{KL}(q(z|x)∥p(z|x)) \end{array}$$

A positive value is always returned by the Kullback–Leibler divergence. As a result, the inequality that results is correct at all times (refer to Equation 15).


(15) $$\begin{array}{l} log\;p\left(x\right)\geq E_{z\sim q\left(z|x\right)}\left[log\;p\left(x|z\right)\right]-D_{KL}\left(q\left(z|x\right)∥p\left(z\right)\right)=ELBO \end{array}$$

This concept is referred to as effective lower bound (ELBO). Since this inequality is always valid, increasing the ELBO value leads to an increase in the decoder's log-marginal likelihood. Calculating the loss function of the VAE by multiplying the right-hand side of the equation by a negative value is possible. The loss function that is used to calculate the training of the convolutional variational autoencoder model is given by Equation (16).


(16) $$\begin{array}{l} L_{VAE}=-E_{z\sim q(z|x)}[log\;p(x|z)]+D_{KL}(q(z|x)∥p(z))\\ \;=L_{Reconstruction}+L_{KD} \end{array}$$

Where *L*_*Reconstruction*_ represents the reconstruction loss, which is the autoencoder's cross-entropy calculated using input and output data, and *L*_*KD*_ indicates Kullback divergence regularizer value, which becomes lower as the inferred probability distribution gets closer to a zero-mean Gaussian distribution.

## 3 Results

### 3.1 Computation of FC and ISFC matrices

For decoding of the cognitive states, we performed analysis in two ways: (a) including the complete dataset, and (b) considering age-wise sub-groups, i.e., 3-yrs, 4-yrs, 5-yrs, 7-yrs, 8–12 yrs, 3–5 yrs, 7–12 yrs, and adults. The dataset was divided into subgroups to check the effect of age on the model's performance. Literature informs that Richardson et al. (2018), networks are not adequately segregated from each other in early childhood. While we considered a dataset that included data from 3-yr old children, it remained a question of whether age is a dependent parameter on the model's performance. To overcome above mentioned hypothesis, we extracted BOLD signal timecourses from 12 ROIs as listed in Table 1 (6 ToM ROIs and 6 pain ROIs) from the mentioned 10 time windows with peak activation. FC matrices of size 12*12 were constructed by calculating Pearson's correlation for each individual. Similarly, we calculated ISFC matrices of size 12*12 for ToM and pain networks. To validate our results, we performed multiple one-sample t-tests for each connection with a *p*-value <0.01 and applied FDR correction.

### 3.2 Decoding of cognitive states using Ex-stGCNN model

For decoding the cognitive states, we implemented the proposed Ex-stGCNN model. We used FC and ISFC matrices as separate feature sets to check whether ISFC, a stimulus-driven feature set, could decode states better than a non-specific feature set. The considered datasets could suffer from some issues: (a) improper network segregation at an early childhood stage, and (b) activation of other brain networks such as the visual networks and the default-mode network during naturalistic-stimuli watching. To clarify how the activation of other networks at the same time could affect the model's performance, we performed an analysis on the whole brain. We compared the results of decoding cognitive states using 12 ROIs (ToM and Pain networks) with decoding using the whole brain FCs and ISFCs. We split the data into ratio of 80:20 (Kahloot and Ekler, 2021; Muraina, 2022). We carried out the analysis using the ratio of 80:20 and reported detailed results using the same ratio. We also compared the performance of traditional existing models like MVPA (Haxby et al., 2001), LSTM-RNN (Li and Fan, 2019), and CNN (Wang et al., 2020) with the proposed model. We found better results from the proposed model than any other existing models (Refer to Table 5).

**Table 5:** Table shows the comparison between performance of traditional models and proposed model on complete dataset.

**Feature set (Including 12 ROIs)**	**Sr. No**.	**Models**	**Accuracy**	**F1-Score**
**FC**	1.a	MVPA	73.51%	0.74
	1.b	LSTM-RNN	77.80%	0.76
	1.c	CNN	79.21%	0.80
	1.d	Ex-stGCNN (Node2Vec)	**85.68%**	**0.84**
**ISFC**	2.a	MVPA	79.23%	0.70
	2.b	LSTM-RNN	83.55%	0.84
	2.c	CNN	85.92%	0.86
	2.d	Ex-stGCNN (Node2Vec)	**94.35%**	**0.95**

The proposed model outperforms the existing models. The bold values represent the best performance using the proposed model.

#### 3.2.1 Using FC matrices as feature set

**Figure 6:**
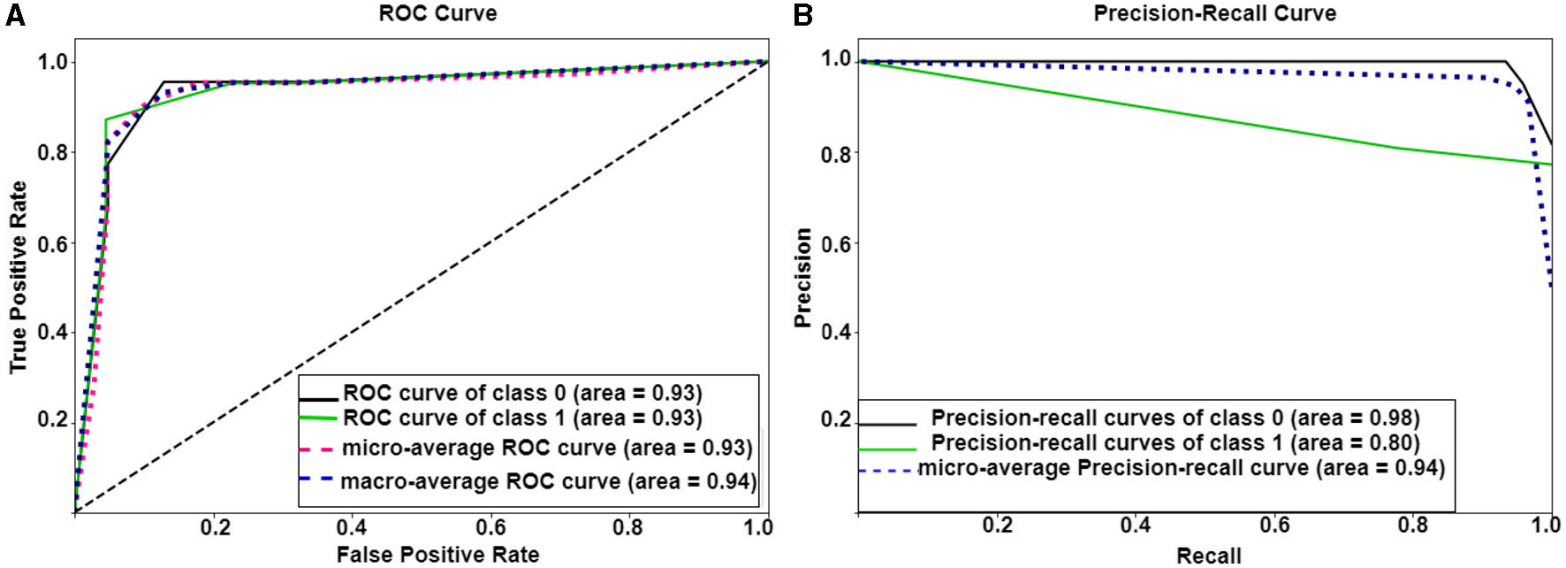
Performance of Ex-stGCNN model: **(A, B)** show ROC and precision-recall curves of Ex-stGCNN model using ISFC matrices.

1. **Analysis on complete dataset:** To perform an ablation study on the proposed model, We implemented two different node embedding algorithms, i.e., Walklets and Node2Vec, as well as tuned the model using different batch sizes. Our observations indicated that Node2Vec outperformed Walklets. Using the Node2Vec algorithm for 3D-Convolutional layers, we achieved an average accuracy of 85% with an F1-score of 0.87 for 12 ROIs, while for the whole brain in the same scenario, we achieved 80% accuracy with an F1-score of 0.79. When Walklets were employed for 3D-convolutional layers, we attained an average accuracy of 78% with an F1-score of 0.80 for 12 ROIs and 73% accuracy with an F1-score of 0.72 for the whole brain. Our results suggest that GCNN with 3D convolutional layers performs better in decoding cognitive states than 2D or 1D convolutional layers, as indicated in Table 6. We validated our results using five-fold cross-validation and achieved an average accuracy of 75% with an F1-score of 0.76. We also implemented leave-one-out method and achieved an average accuracy of 78% with F1-score of 0.75.
2. **Analysis on age-wise sub-groups:** Additionally, we analyzed age-wise subgroups to check effect of age on the model's performance (Refer to Figure 6). We achieved the lowest accuracy of 50% with an F1-score of 0.48 for the 3-yrs age group using Walklets, and the pattern was the same for 4-yrs. We observed a change in the model's performance from the 7-yrs age group with an accuracy of 68% with an F1-score of 0.69 for 12 ROIs and achieved the highest accuracy for adult groups with an average accuracy of 85% with an F1-score of 0.84. We validated our results using five-fold cross-validation and achieved an average accuracy of 68% with an F1-score of 0.67. We found an average accuracy of 69% with F1-score of 0.71 using leave-one-out method.

**Table 6:** Table shows the performance of proposed Ex-stGCNN model using ISFC and FC matrices.

**Feature set**	**Convolutional layer**	**Filters**	**Walklets (whole brain)**	**Walklets (12 ROIs)**	**Node2Vec (whole brain)**	**Node2Vec (12 ROIs)**
-	-	-	**Accuracy**	**Accuracy**	**Accuracy**	**Accuracy**
**FC**	1D	16	58.15%	61.50%	59.71%	60.25%
	2D	16,32	65.60%	68.36%	68.48%	75.23%
	3D	16,32,64	73.85%	78.72%	80.92%	85.68%
**ISFC**	1D	16	63.45%	65.66%	65.87%	69.70%
	2D	16,32	75.61%	79.01%	82.86%	88.21%
	3D	16,32,64	82.46%	92.57%	85.75%	**94.35**%

We found more accurate results using ISFC matrices as compared to FC matrices. The bold values represent the best performance using the proposed model.

For explainability, we applied SHAP(Shapley Additive exPlanations), which provided the extent to which each input feature contributed to the prediction. We computed the median of feature scores and identified ROIs that contributed the most to classification. We observed that bilateral Temporoparietal Junction (LTPJ and RTPJ), Ventromedial Prefrontal Cortex (vmPFC), Left Interior Insula, and Bilateral Middle Frontal Gyrus (LMFG and RMFG) contributed most to the prediction (Refer to Figure 6).

#### 3.2.2 Using ISFC matrices as feature set

**Figure 7:**
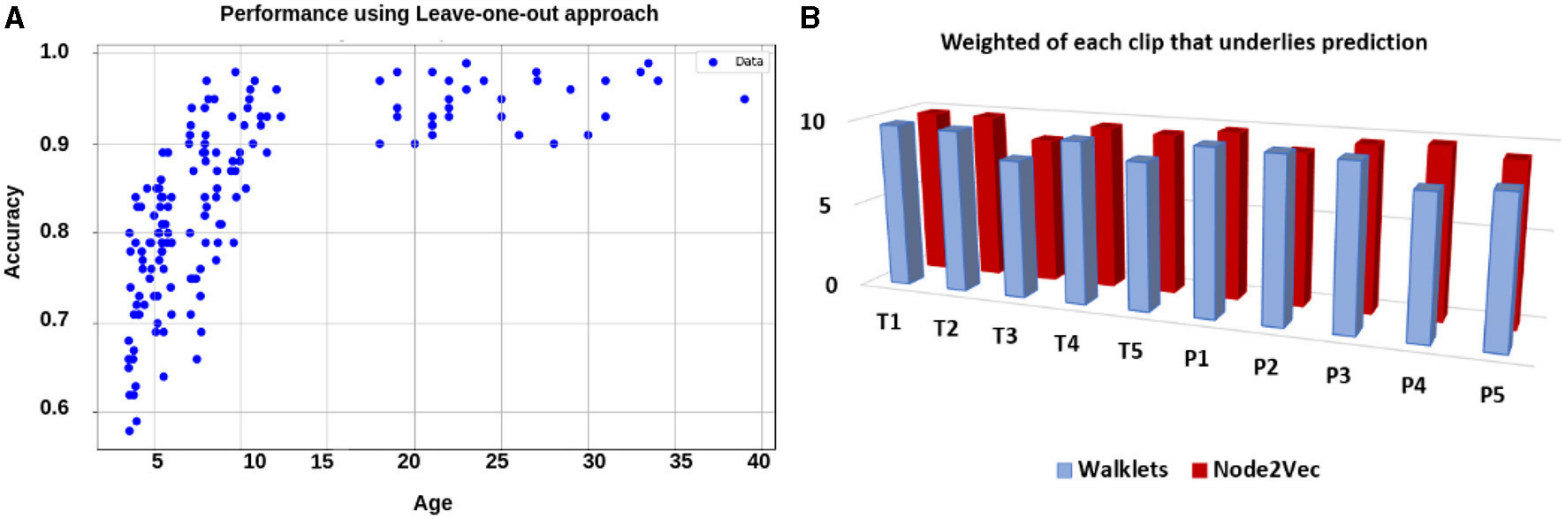
Performance using leave-one-out approach: **(A)** shows performance in terms of accuracy for each individual. It shows average accuracy for all 10 time-course for each participant. The results suggested that as age increases, the performance of the model also increases. **(B)** shows the weight of each time-course in the prediction.

1. **Analysis on complete dataset:** We hypothesized that stimulus-driven measures could better predict the brain state. To test our hypothesis, we calculated ISFC matrices and trained the model. During testing, we achieved the highest accuracy of 94% with an F1-score of 0.95 for 12 ROIs and 85% with an F1-score of 0.87 for the whole brain using Node2Vec for 3D-convolutional layers (Refer to Table 6). In contrast, we obtained an average accuracy of 92% with a 0.93 F1-score for 12 ROIs and 82% accuracy with a 0.83 F1-score for the whole brain using Walklets for 3D-convolutional layers. Hence, our hypothesis was correct: ISFC measures provided better results compared to FC measures. To validate the results, we conducted a five-fold cross-validation and achieved an average accuracy of 91% with an F1-score of 0.91. Using the leave-one-out method, we achieved an average accuracy of 93% with an F1-score of 0.93. We observed that false-positive cases belonged to the early childhood age group, i.e., 3-yrs and 4-yrs as shown in Figure 7.
2. **Analysis on age-wise sub-groups:** We analyzed age-wise sub-groups and achieved better results using ISFC matrices. We achieved the best accuracy of 74% with an F1-score of 0.75 for 12 ROIs for the 3-yr age group. This proves that despite incomplete network segregation during early development, ISFC measures could still predict states to a reasonable extent.

Using the SHAP explainability method, we observed that bilateral Temporoparietal Junction (LTPJ and RTPJ), Posterior Cingulate Cortex (PCC), Right Interior Insula, and Bilateral Middle Frontal Gyrus (LMFG and RMFG) contributed most to the prediction of cognitive state.

### 3.3 Prediction of individual performance on false-belief tasks

**Figure 8:**
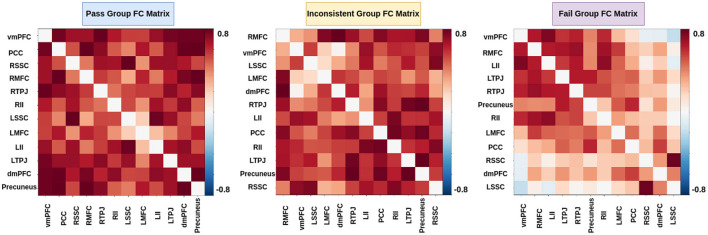
Functional connectivity between ToM and pain networks for false-belief task-based pass, fail, and inconsistent groups. Pass group showed stronger connectivity between ToM and pain network, whereas inconsistent group showed moderate connectivity, and fail group showed weaker connectivity.

**Figure 9:**
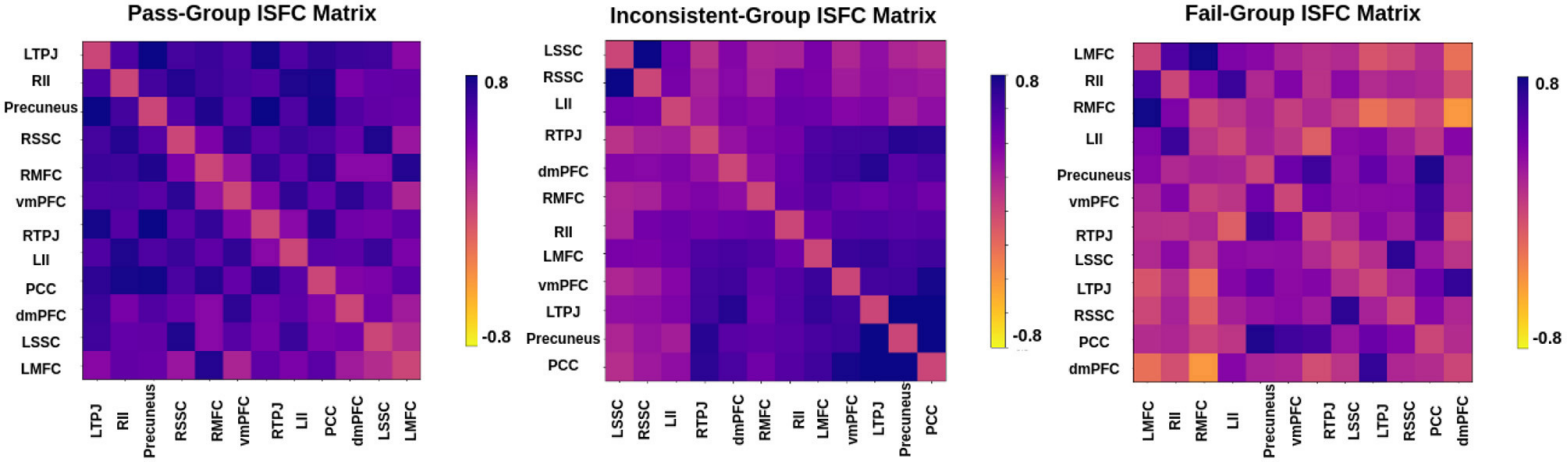
Inter-Subject Functional connectivity between ToM and pain networks for the false-belief task-based pass, fail, and inconsistent groups. The FC and ISFC matrices show similar patterns, i.e., stronger connectivity for the pass group, moderate connectivity for the inconsistent group, and weaker connectivity for the fail group.

In the literature Li et al. (2019a,b) and Finn and Bandettini (2021), an association between brain signals and behavioral scores has been found. We hypothesized that functional connectivity and inter-subject functional connectivity between selected brain regions could predict individual performance on false-belief tasks. To check our hypothesis, we extracted FC and ISFC matrices from selected brain regions for each individual. We observed stronger connectivity for the false-belief task-based pass group, whereas moderate connectivity for an inconsistent group and weaker connectivity for the fail group using FC and ISFC matrices (Refer to Figures 8, 9). To validate our results, we performed multiple one-sample t-tests, one for each connection, with a *p*-value <0.01, and applied FDR correction. Here, we referred to stronger connectivity if the correlation between the regions >0.5, if correlation ≈0.5, then it indicated moderate connectivity, and if correlation <0.5, then it is referred to as weaker connectivity.

For prediction of individual performance on false-belief tasks, we trained multiple ML and DL models, for example, decision tree, random forest, SVM, and proposed Ex-stGCNN. We trained the mentioned models in 3 ways: (a) using all 12 ROIs, (b) using 8 ROIs that contributed most (dominant ROIs) in decoding of cognitive state, and (c) using only 6 ToM ROIs. We divided dataset into 80:20 ratios and provided FC and ISFC matrices seperately as input to train the model. The mentioned models were not able to give accurate results as reported in Table 7. To overcome the limitation of mentioned models, we proposed an Ex-Convolutional VAE model to predict individual performance on false-belief tasks and categorized participants into pass, inconsistent, and fail groups. Using FC matrices, we achieved 90% accuracy with F1-score of 0.91 using 12 ROIs, 84% accuracy with F1-score of 0.83 using eight dominant ROIs, and 80% accuracy with 0.79 F1-score using six ToM ROIs. To validate our results, we performed five-fold cross-validation and achieved an average accuracy of 87% with F1-score of 0.88. We also achieved average accuracy of 85% with F1-score of 0.84 using leave-one-out method. We also tried 1D convolutional and achieved 81% accuracy with 0.80 F1-score using 12 ROIs, 73% with 0.74 F1-score using 8-dominant ROIs, and 66% accuracy with F1-score of 0.67 using six-Tom ROIs using FC matrices. Whereas, using ISFC matrices, we achieved 93.5 % accuracy with F1-score 0.94 using 12 ROIs, 89% accuracy with F1-score 0.87 using eight dominant ROIs, and 83% accuracy with F1-score 0.82 using ToM ROIs. We also validated our results using five-fold cross-validation and achieved an average of 90% accuracy with an F1-score of 0.89. We achieved average accuracy of 92% with F1-score of 0.91 using leave-one-out method.

**Table 7:** Table shows the comparison between the performance of multiple models and the proposed model for predicting individual performance on false-belief tasks.

**Feature set (Including 12 ROIs)**	**Sr. No**.	**Models**	**Accuracy**	**F1-Score**	**Precision**	**Recall**
**FC**	1.a	Decision Tree	69%	0.68	0.67	0.69
	1.b	Random-Forest	65%	0.66	0.62	0.63
	1.c	SVM	61%	0.62	0.59	0.60
	1.d	Proposed Ex-stGCNN	84%	0.85	0.83	0.81
	1.e	Proposed Ex-Convolutional VAE	**90%**	**0.91**	**0.89**	**0.87**
**ISFC**	2.a	Decision Tree	72%	0.71	0.69	0.70
	2.b	Random Forest	70%	0.72	0.71	0.69
	2.c	SVM	65%	0.67	0.64	0.61
	2.d	Proposed Ex-stGCNN	89%	0.90	0.87	0.85
	2.e	Proposed Ex-Convolutional VAE	**93.5%**	**0.94**	**0.91**	**0.90**

The proposed Ex-Convolutional VAE model outperforms as compared to Ex-stGCNN and other existing models. The bold values represent the best performance using the proposed model.

## 4 Discussion

We identified interpretable dynamic brain features using a novel stGCNN model that accurately decodes time-locked stimulus-driven cognitive states during ongoing movie scene experience, even in children as young as 3-yrs. Children (*n* = 122, 3–12 yrs) and adults (*n* = 33) watched a short, engaging animated movie while undergoing fMRI. The movie highlights the characters' bodily sensations (often pain) and mental states (beliefs, desires, emotions) and is a feasible experiment for young children. We model learned latent dynamic interactions among distributed brain regions of interest without *ad hoc* feature engineering, achieving high classification accuracies in cross-validation analysis in a naturalistic paradigm. Decoding and mapping cognitive states of the human brain is an exciting area of research for learning context-specific and independent cognitive architectures and their developmental differences. However, identifying and mapping cognitive states in early childhood and late adolescence is challenging (Simony and Chang, 2020) as extant literature (Astington and Edward, 2010; Richardson et al., 2018) suggests that brain networks are not adequately segregated in the early childhood stage (as early as 3-yrs). Young children's brain development and cognitive abilities undergo substantial transformations during the initial years of their lives (Schult and Wellman, 1997; Schulz et al., 2007; Cohen et al., 2011; Richardson et al., 2018). Deep learning models showed great success in decoding and mapping diverse cognitive states of the human brain (Wang et al., 2020). Despite this exciting development, existing models (Zhang et al., 2021, 2023; Ye et al., 2023) suffer from an issue of low accuracy and explainability due to their internal architecture and feature extraction technique. Also, the existing models (Zhang et al., 2021, 2023; Saeidi et al., 2022; Ye et al., 2023) were tested out in adult data when brain networks are fully matured. Identifying the most effective features that could categorize the relationship between complex naturalistic stimuli and the associated brain activity in children remains unexplored. Moreover, it is pertinent to ask how to design deep learning architecture that could examine the complex representation of brain networks during early development.

### 4.1 Decoding of cognitive states using Ex-stGCNN model

Previously the proposed methods, i.e., multivariate pattern analysis (MVPA) (Haxby et al., 2001), RNN-based method (Li and Fan, 2019), and CNN-based (Wang et al., 2020) showed significant results in decoding multiple cognitive states from fMRI signals of the brain without any burden for handcrafted features. Among previously proposed methods, RNN with LSTM, a deep learning method for sequence modeling, ignores spatial information within the input data (Sepp Hochreiter, 1997). The 2D CNN-based methods cannot encode the 3D nature of fMRI data. Thus, Meszlényi et al. (2017) and Li and Fan (2019) methods require functional network-based features as inputs. Previous studies have also proposed a deep learning framework based on the graph convolutional neural networks (GCNNs) presented to enhance the decoding performance of raw EEG signals during different types of motor imagery (MI) tasks while cooperating with the functional topological relationship of electrodes. Based on the absolute Pearson's matrix of overall signals, the graph Laplacian of EEG electrodes is built up. The GCNs-Net constructed by graph convolutional layers learns the generalized features. The following pooling layers reduce dimensionality, and the fully connected SoftMax layer derives the final prediction. The introduced approach has been shown to converge for both personalized and group-wise predictions (Hou et al., 2022). Interestingly, several recent works have focused on identifying individual differences and discovering neurological biomarkers using a GCNN framework to analyze functional magnetic resonance images (fMRI) (Li et al., 2021; Saeidi et al., 2022).

In this study, we proposed a graph-based explainable brain decoding model that combines information on the dynamics of the brain's distributed networks. Here, we designed an Explainable spatiotemporal Connectivity-based Graph-Convolutional Neural Network (Ex-stGCNN) model to decode cognitive states that could represent complex topological relationships and interdependencies between data. We have used stGCNN model using 12 specified ROIs of interest (ROIs) [which included bilateral Temporoparietal Junction (LTPJ and RTPJ), Posterior Cingulate Cortex (PCC), Ventral and Dorsal-medial Prefrontal Cortex (vmPFC and dmPFC), and Precuneus] and ROIs from pain network [which included bilateral Middle Frontal Gyrus (LMFG and RMFG), bilateral Interior Insula, and bilateral Secondary Sensory Cortex (LSSC and RSSC)] based on previous work tracking development in 3–12 yrs old using the same stimuli (Richardson et al., 2018). We also trained the stGCNN model at the whole brain level. Our results revealed simultaneous activation of other brain networks, e.g., Visual Network, DMN, and FPN. stGCNN could not accurately decode task-activated states in children and adolescents in the whole brain analysis, highlighting the model's specificity. The simultaneous activation of multiple brain networks may account for the less accurate results obtained in the whole brain analysis compared with the accuracy achieved when specifically implementing decoding ToM and pain networks. We observed that the Node2Vec node embedding method was giving more accurate results as compared to the Walklets node embedding method in the developmental age group dataset. We also observed the effect of age on the model's performance, i.e., we were getting better results from 5 years onwards. To validate the results and check the performance of the model on an individual level, we also used five-fold cross-validation and leave-one-out methods. We found better results using the leave-one-out method; data splitting might be the case that led to an increase in performance. While the leave-one-out method trains on the entire dataset except for one sample at a time, five-fold cross-validation trains on only a subset of the data in each fold. This difference in training data distribution could affect the model's ability to generalize. We observed that most of the false-positive predictions belonged to early developmental age groups, i.e., 3-yrs, 4-yrs using the leave-one-out method.

### 4.2 Feature identification and brain fingerprinting using Ex-stGCNN

A challenge of applying any graph neural network models to neuroimaging research is the black box characteristic of this approach: No one knows exactly what the graph convolutional network is doing. A model might achieve high levels of decoding accuracy but provide no insight into which features are important for decoding or whether the features are neurobiologically interpretable in the context of empirical evidence based on GLM-based or reverse correlation analysis carried out on ToM and Pain ROIs BOLD time-series signals by previous work (Richardson et al., 2018). Further, the network segregation and activation of other networks could affect the model's performance at a certain level (Li and Fan, 2018, 2019; Albouy et al., 2019; Gao et al., 2019; Wang et al., 2020; Cao et al., 2021).

**Figure 10:**
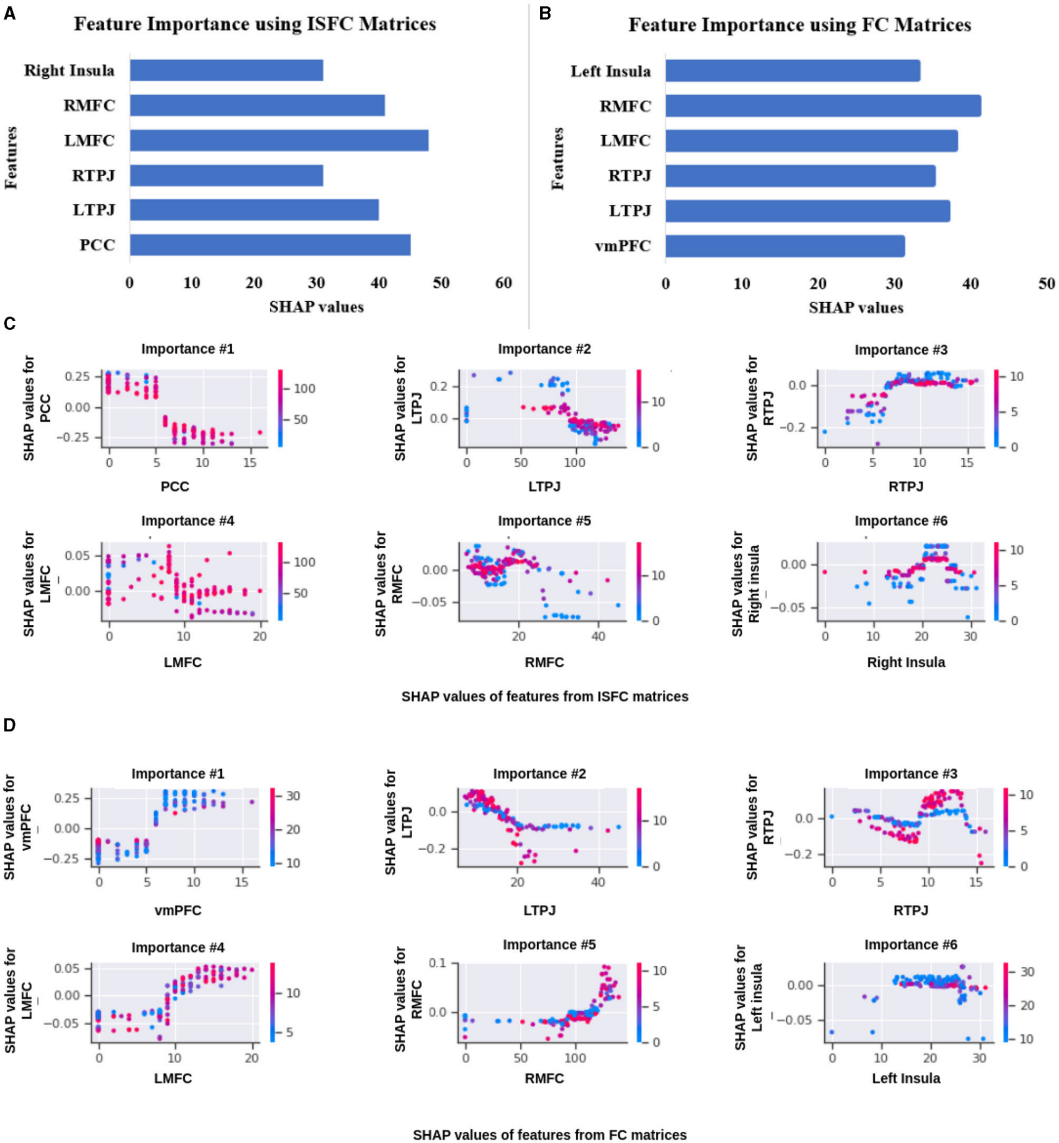
Feature importance. **(A, B)** show brain regions that contributed most to the prediction using ISFC and FC matrices. We extracted three dominating ToM regions and three dominating pain regions. **(C, D)** show a relationship between feature values and the impact they have on the model's predictions. There is an overlap between the regions identified by ISFC and FC matrices.

**Figure 11:**
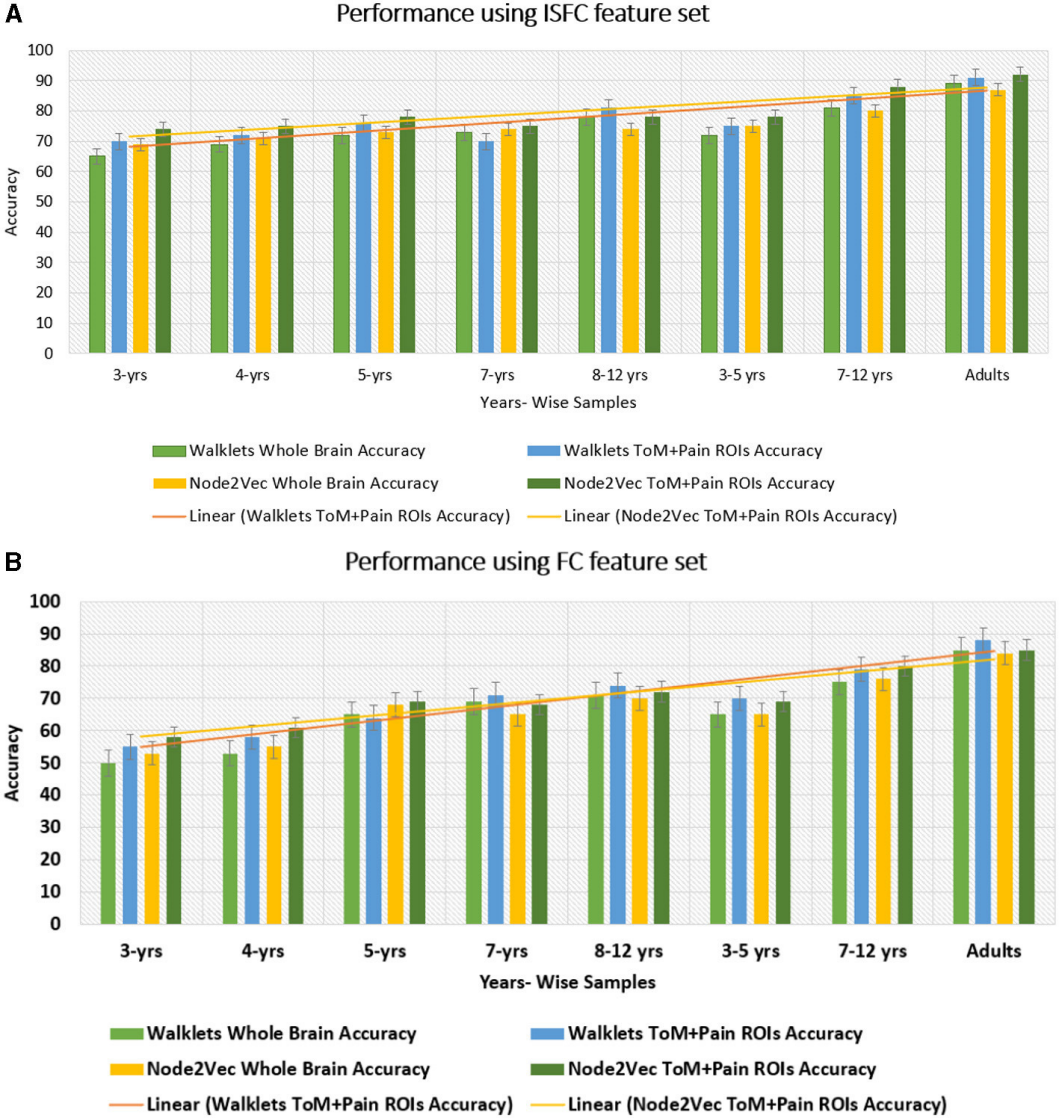
Performance of GCNN model on Age-wise sub-groups: **(A)** shows accuracy of proposed Ex-stGCNN model on sub-groups using ISFC matrices. **(B)** shows accuracy using FC matrices. Our results suggest that we can achieve considerable performance using ISFC matrices at early childhood stage. In sub-group analysis, both Walklets and Node2Vec node embedding methods performed approximately the same.

Using a SHAP approach, our graph learning model allowed us to identify and rank brain connectivity features that distinguish different decoding model performances as reported in Figures 10, 11). Furthermore, our predictive features identify the brain fingerprints, which index individual differences and the differential contribution of different brain areas to the Decoding of cognitive states and predict individual performance during the false-belief task.

For the explainability of the proposed model, we implemented the SHAP approach. We identified three dominant brain regions from ToM and three dominant brain regions from the pain functional network based on both FC and ISFC matrices. We identified that, on average, bilateral Temporoparietal Junction (LTPJ and RTPJ), Ventromedial Prefrontal Cortex (vmPFC), Left Interior Insula, and Bilateral Middle Frontal Gyrus (LMFG and RMFG) contributed most to the prediction using the FC feature set. In contrast, bilateral Temporoparietal Junction (LTPJ and RTPJ), Posterior Cingulate Cortex (PCC), Right Interior Insula, and Bilateral Middle Frontal Gyrus (LMFG and RMFG) contributed most to the prediction using the ISFC feature set.

Our study represents a significant departure from previous studies by directly targeting spatiotemporal stimulus-driven feature sets, i.e., ISFC. Our results showed that even in the early age groups 3-yrs and 4-yrs, ISFC matrices could track stimulus-induced dynamic spatiotemporal brain activation patterns. Notably, the stGCNN model achieved high state decoding accuracy despite age differences, and the accuracy levels were considerably higher than those obtained using conventional methods implementing MVPA, LSTM-RNN, and different versions of CNN models (summary in Table 5 and Figure 11). Our stGCNN-based feature detection analysis identified the bilateral Temporoparietal Junction (LTPJ and RTPJ), Posterior Cingulate Cortex (PCC), Right Interior Insula, and Bilateral Middle Frontal Gyrus (LMFG and RMFG), which anchor the mentalization network important for differentiating social and non-social stimuli, DMN, as brain areas whose dynamic properties most clearly distinguished the individual differences in dynamic patterns. Crucially, these features were observed in the children and replicated in the adults, further attesting to the robustness of our findings. Aberrancies in nodes that anchor the mentalization, self-processing networks, and their static and dynamic functional interactions contribute substantially to the differential functional integration of information and belief about the self and others in the context of the stimuli used in this study in line with the previous findings. This further suggests that the proposed Ex-stGCNN model can be used as a research tool to provide important insights about task/cognition-specific brain connectivity and dynamics.

### 4.3 Prediction of individual performance in false-belief taks

**Figure 12:**
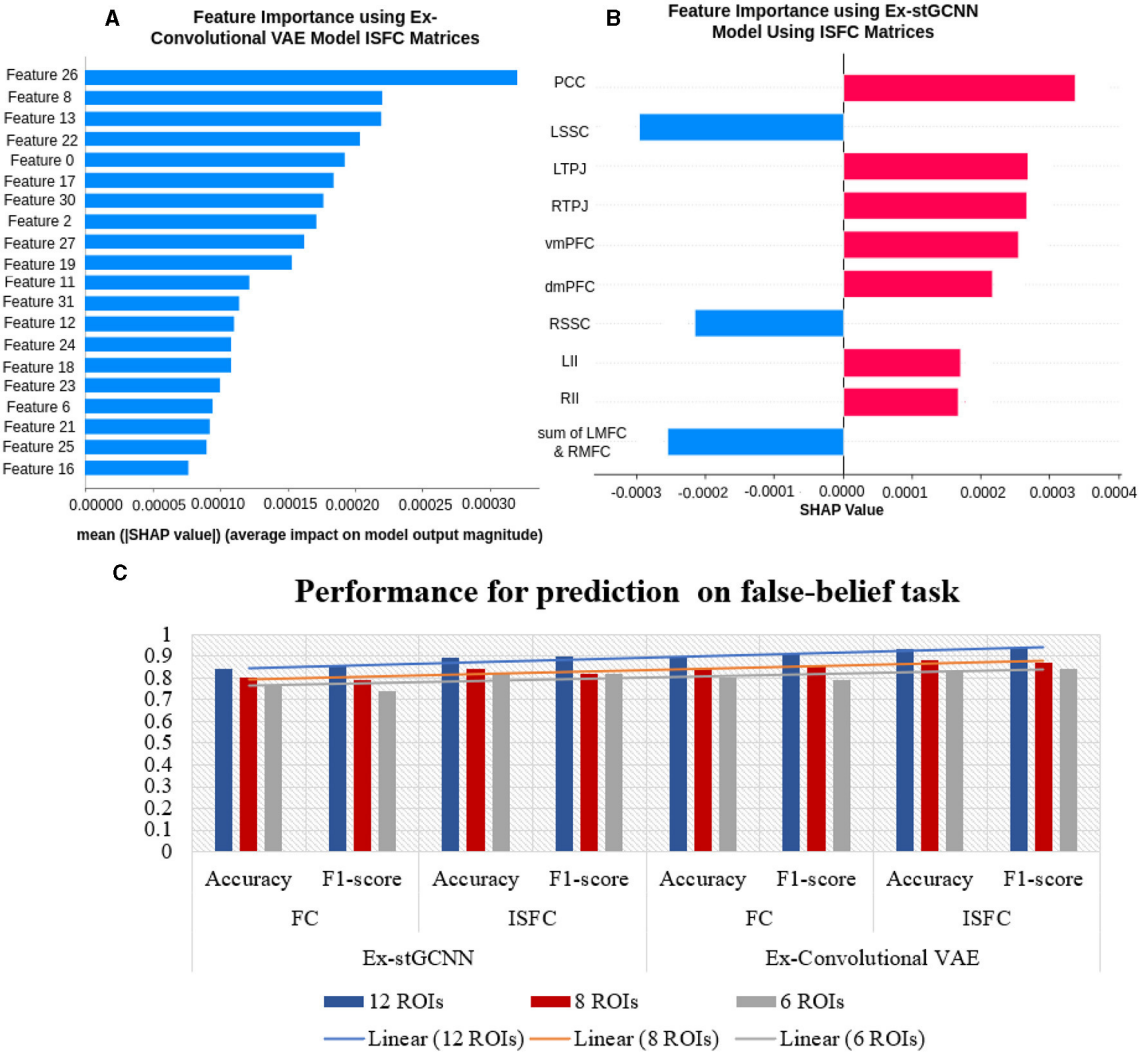
Performance for prediction of false-belief task: **(A)** Shows importance of features and their SHAP values using ISFC matrices for the proposed Ex-Convolutional VAE model. **(B)** shows feature importance using ISFC matrices for proposed Ex-stGCNN model **(C)** Shows overall performance.

The final challenge we addressed here was to uncover neurobiologically interpretable features of inter-subject brain connectivity patterns to predict individual performance in a false-belief task. Previous studies have shown rTPJ is frequently associated with different capacities to shift attention to unexpected stimuli (reorienting of attention) and to understand others' (false) mental state [theory of mind (ToM), typically represented by false belief tasks]. Many studies further suggest that two dominant subregions, posterior rTPJ seem exclusively involved in the social domain, and anterior rTPJ is involved in both attention and ToM, conceivably indicating an attentional shifting role of this region (Krall et al., 2015; Igelström and Graziano, 2017). A recent study (Ganesan et al., 2022) reported that behavioral measures related to visual stimuli could influence the models performance in classifying between rest and task states using static and dynamic functional connectivity. We hypothesized that neurobiologically interpretable brain features of FC and ISFC between specified brain regions could effectively predict individual performance in false-belief tasks. To test our hypothesis, we implemented multiple models in which FC and ISFC matrices were subjected as input as listed out in Table 7. We were getting moderate results. To overcome the limitations of tested models, we designed an Explainable Convolutional Variational Autoencoder (Ex-Convolutional VAE) model. We observed stronger correlations for the false belief task-based pass group, moderate connectivity for an inconsistent group, and anticorrelations for the fail group amongst the 12 selected ROIs as mentioned in Figures 8, 9, 12). Here, we trained the model using the FC and ISFC matrices separately for each subject. We observed that FC and ISFC between ToM and pain networks (identified features) affected the model's performance, i.e., improvement in the model's performance compared with when only FC and ISFC within the ToM regions was considered. Across the 5-fold cross-validation analysis, the bilateral Temporoparietal Junction (LTPJ and RTPJ), Posterior Cingulate Cortex (PCC), Right Interior Insula, and Bilateral Middle Frontal Gyrus (LMFG and RMFG) are the only brain regions whose features strongly predicted the individual performance in the false-belief task.

Interestingly, we also found that individuals who mostly failed in the false-belief task belonged to the 3-yrs old and 4-yrs old age groups. This is largely consistent with previous neuroimaging findings, which suggest that brain regions involved in ToM in adulthood already constitute a distinct network in 3-yr old children. The ToM network gradually becomes more integrated and distinct from other networks over the next decade. Similarly, the response time course in the ToM network in response to a social movie is strongly positively correlated, even between 3-yr olds and adults. The time course and peak event responses show gradual continuous development over childhood. Focusing specifically on 3-5 yrs old children, the neural responses to social movies in children who systematically fail versus pass explicit false-belief tasks were similar: there were no differences in the magnitude of response to the five ToM events (Table 2) identified using reverse correlation analyses (Richardson et al., 2018), as indicated here by observed stronger correlation between ToM and the pain network in the passers and in contrast, anticorrelations in the fail group suggesting between network correlation is necessary for performing well in the mentalization task.

### 4.4 Limitations and future scope

Although the proposed framework provided a promising avenue for decoding cognitive states and predicting false-belief performance in developmental dataset, the study had some limitations. The dataset comprised only 155 participants (age range 3–12 yrs), in which some age ranges did not have enough no. of participants, for example, the adults group (age range 13–39), and for 6-yrs age, there were no participants. So, we could not treat data in a continuous manner. The proposed Ex-stGCNN was unable to capture brain dynamics for the early childhood age range, i.e. 3-yrs and 4 yrs which led to decreased performance in decoding of cognitive states. The proposed model was also not able to give accurate predictions in whole brain analysis due to the activation of other brain regions during visual stimuli watching. We used two different models, i.e., Ex-stGCNN and Convolutional-VAE models for prediction of performance in false-belief tasks. We found better results using the Ex-Convolutional VAE model that opened the door to examine the limitation of the Ex-stGCNN model for prediction of performance for false-belief tasks. In the future, we will try to address the mentioned limitations. We will also try gender differences in the decoding of states.

## 5 Conclusion and future aspects

The study aimed to propose a framework that can decode higher-order brain states and associated cognition using short time-courses brain signals for developmental age group dataset collected from single session recordings without using feature engineering and which can also predict individual performance on false-belief tasks and categorize them in pass, fail, and inconsistent subject groups. We trained the model using ISFC and FC matrices separately and achieved 94% accuracy using ISFC matrices and 85% using FC matrices. We also analyzed age-wise subgroups to check the effect of age on the model's performance. Due to incomplete network segregation at the early childhood stage, the model gives lower accuracy for early age groups, i.e., 3-yrs and 4-yrs, as for the 5-yrs and above. We used the SHAP approach to determine the brain fingerprints that contributed most to the prediction. We show that our proposed architecture did perform superior to traditional fMRI decoding, RNN, and CNN-based models for complex cognitive states during the naturalistic experience in individuals of early childhood and pre-adolescence, even with short event time-courses and small datasets. To predict false-belief task-based pass, fail, and inconsistent groups, we proposed an Ex-Convolutional VAE model and achieved 90% accuracy using FC matrices and 93.5% using ISFC matrices. We validated our results using five-fold cross-validation. our results suggested that stimulus-driven features such as ISFC could better capture brain states even in the early developmental age-group data.

## Data availability statement

Publicly available datasets were analyzed in this study. This data can be found here: the fMRI and behavioral data collected and analyzed during the current study are available through the OpenfMRI project (https://openfmri.org/; Link: https://www.openfmri.org/dataset/ds000228/ 10.5072/FK2V69GD88). The ToM behavioral battery is additionally available through OSF (https://osf.io/G5ZPV/; 10.17605/OSF.IO/G5ZPV; ARK: c7605/osf.io/g5zpv). The corresponding author welcomes any additional requests for materials or data.

## Ethics statement

The studies involving humans were approved by child and adult participants were recruited from the local community. All adult participants gave written consent; parent/guardian consent and child assent were received for all child participants. Recruitment and experiment protocols were approved by the Committee on the Use of Humans as Experimental Subjects (COUHES) at the Massachusetts Institute of Technology. The studies were conducted in accordance with the local legislation and institutional requirements. Written informed consent for participation in this study was provided by the participants' legal guardians/next of kin.

## Author contributions

KB: Conceptualization, Data curation, Formal analysis, Investigation, Methodology, Resources, Software, Validation, Visualization, Writing – original draft, Writing – review & editing. AA: Formal analysis, Methodology, Software, Writing – review & editing. RB: Investigation, Methodology, Project administration, Supervision, Validation, Writing – original draft, Writing – review & editing. DR: Conceptualization, Data curation, Formal analysis, Funding acquisition, Investigation, Methodology, Project administration, Resources, Software, Supervision, Validation, Visualization, Writing – original draft, Writing – review & editing.
